# Correlates of physical activity in a population-based sample of kidney cancer survivors: an application of the theory of planned behavior

**DOI:** 10.1186/1479-5868-9-96

**Published:** 2012-08-06

**Authors:** Linda Trinh, Ronald C Plotnikoff, Ryan E Rhodes, Scott North, Kerry S Courneya

**Affiliations:** 1 Faculty of Physical Education and Recreation, University of Alberta, Edmonton, Edmonton, Alberta, T6G 2H9, Canada; 2 Priority Research Centre in Physical Activity and Nutrition, The University of Newcastle, Callaghan, NSW, 2308, Australia; 3 Behavioral Medicine Laboratory, Faculty of Education, University of Victoria, PO Box 3010 STN CSC, Victoria, V8W 3N4, British Columbia, Canada; 4 Department of Medicine, Cross Cancer Institute, 11560 University Ave., T6G 1Z2, Edmonton, Alberta, Canada

**Keywords:** Exercise, Motivation, Social cognitive models, Correlates

## Abstract

**Background:**

Over half of kidney cancer survivors (KCS) are completely inactive and only a quarter are meeting physical activity (PA) guidelines. This highlights the need to identify and understand the determinants of PA in this understudied population. The purpose of this study is to determine the social cognitive correlates of PA intention and behavior in KCS using the Theory of Planned Behavior (TPB).

**Methods:**

All 1,985 KCS diagnosed between 1996 and 2010 in Alberta, Canada were mailed a self-report survey that consisted of the Godin Leisure Time Exercise Questionnaire and standard TPB items for intention, planning, perceived behavioral control (PBC), affective and instrumental attitudes, and descriptive and injunctive norms. Standard demographic and medical variables were also collected.

**Results:**

Completed surveys were received from 703 of 1,654 (43%) eligible KCS. The TPB was tested using structural equation modelling and demonstrated an adequate-to-good fit to the data [χ² = 256.88, p < .001; TLI = 0.97; CFI = 0.98; RMSEA = 0.06, 90% CI = 0.05-0.06].

There were significant pathways to PA from PBC (ß = 0.18, p = 0.02), planning (ß = 0.22, p < 0.01), and intention (ß = 0.31, p < 0.01); and to planning from intention (ß = 0.81, p < 0.01). In addition, there were significant model pathways to intention from instrumental attitude (ß = 0.28, p = 0.03), descriptive norm (ß = 0.09, p = 0.01), and PBC (ß = 0.52, p < 0.01). Overall, the TPB accounted for 69%, 63%, and 42% of the variance in intention, planning and PA, respectively.

**Conclusion:**

The TPB appears to be a useful model for explaining PA in KCS. All TPB constructs except injunctive norm and affective attitude were useful for explaining intention with PBC emerging as the largest correlate. Developing PA interventions based on the TPB may be effective in promoting PA in KCS and may lead to important improvements in health.

## Background

Physical activity (PA) improves quality of life (QoL) in cancer survivors [1-3] including kidney cancer survivors (KCS) [4]. Most cancer survivors, however, are not meeting PA guidelines and little is known about the correlates of PA in this population [5,6]. Moreover, the correlates of PA may vary by cancer survivor group [5]. Previous studies have focused on colorectal [7], young adult [8], breast [9], prostate [9], non-Hodgkin lymphoma [10], multiple myeloma [11], endometrial [12], and bladder cancer survivors [13] and have demonstrated important differences in the determinants of PA, but no study to date has focused on KCS. KCS have unique disease and treatment-related factors that may influence the correlates of PA. Since there are numerous demographic and medical differences between survivor groups, it is important to collect data on individual cancer groups, rather than attempt to generalize the results from other cancer populations. In a population-based survey, we previously reported that PA was associated with improved QoL and fatigue in KCS, but only 25% were meeting PA guidelines [4]. Here, we report the correlates of PA in KCS using the Theory of Planned Behavior (TPB) and structural equation modeling (SEM).

The TPB proposes that a person’s intention to perform a behavior is the immediate determinant of that behavior as it reflects the level of motivation a person is willing to exert to perform the behavior [14]. Intention is theorized to mediate the influence of three main constructs on behavior: attitude, subjective norm, and perceived behavioral control (PBC). Attitude reflects a positive or negative evaluation of performing the behavior, and has both instrumental (e.g., harmful/beneficial) and affective (e.g., boring/enjoyable) components. Subjective norm is defined as the perceived social pressure to perform the behavior, and includes both injunctive (e.g., what significant others think the person should do) and descriptive (e.g., what significant others themselves do) components. PBC is an evaluation of how easy or difficult it will be to perform a behavior. Empirical evidence has demonstrated the superiority of the two-component TPB model over the traditional single component model for attitude and subjective norm but not for PBC [15-18]. Moreover, integration of a planning construct into the TPB may be an important pathway for translating intentions into behavior. Furthermore, the TPB also proposes that attitude, subjective norm, and PBC are determined by salient behavioral, normative, and control beliefs [14].

Previous studies in cancer survivors examining the correlates of PA using the TPB have employed multivariate statistical procedures such as path analyses or hierarchical regression, but the process of obtaining this analysis is simply a function of running a series of regressions. This statistical approach does not estimate the overall theoretical model, but instead examines sections of the theoretical model. Therefore, the evaluation of the overall fit of the TPB model to the data cannot be obtained [19]. On the other hand, in our study, we employed SEM to examine the correlates of PA, which is a major advantage over other statistical procedures. The benefit of SEM is the ability to test of the hypothesized relationships among observable and latent variables in the TPB completely and simultaneously [19,20]. Modeling TPB constructs as latent variables allows researchers to take into account measurement error which may influence the relationships in the model [19,20].

The purposes of this study are to: (a) test the utility of the modified TPB (i.e., the inclusion of the planning construct) in KCS, and to determine the most important social cognitive correlates of PA intentions and behavior; (b) determine if the TPB operates equivalently across commonly selected demographic (i.e., age, sex) and medical [i.e. body mass index (BMI), number of comorbidites, months since diagnosis, type of surgery, type of incision, disease stage) variables; and (c) identify the most common behavioral, control, and normative beliefs of KCS. Based on the theoretical tenets of the TPB [14] and previous studies in cancer survivors [7,8,12,13,21], we hypothesized that PBC, affective and instrumental attitude, and descriptive norm would be the most important correlates of PA intentions in KCS. We also hypothesized that intention, PBC, and planning will be the most important correlates of PA. The assessment of whether the TPB operates equivalently across commonly selected demographic and medical characteristics was considered exploratory.

## Methods

### Participants and Procedures

The current study is from a dataset examining PA and health in KCS, where previous analyses included QoL and PA among KCS [4], as well as examining PA preferences among KCS [22]. Ethical approval was obtained through the Alberta Cancer Board Research Ethics Board and the University of Alberta Health Research Ethics Board. The methods of the survey have been reported elsewhere [4]. Briefly, a population-based, cross-sectional, mailed survey of KCS was utilized. Eligibility status included: (a) at least 18 years old, (b) provided written informed consent in English, and (c) diagnosed with kidney cancer. All 1,985 KCS diagnosed between 1996 and 2010 were drawn from the Alberta Cancer Registry. Eligible survivors were mailed a survey package that included: (a) an invitation letter from the registry; (b) a letter from the researchers explaining the study purpose, (c) the survey booklet, and (d) a postage paid return envelope. The survey protocol followed a modified version of the Total Design Method [23] wherein prospective participants were mailed: (a) the initial study package, (b) a postcard reminder 3–4 weeks later to nonresponders, and (c) a second survey package 3–4 weeks later to nonresponders from the initial survey and reminder.

#### Measures

##### Demographic and medical information

Demographic variables were measured using self-report and included age, sex, education level, marital status, annual income, employment status, ethnicity, and height and weight to calculate BMI. Medical variables were also measured using self-report and included time since diagnosis, type of kidney cancer, lymph node involvement, disease stage, previous and current treatments, previous recurrence, current disease status, smoking and drinking status, and comorbidities.

##### Physical activity

A modified version of the Leisure Score Index (LSI) from the Godin Leisure-Time Exercise Questionnaire (GLTEQ), that has been extensively validated [24,25], was used to assess PA. Participants were asked to report their average weekly frequency and duration of light (minimal effort, no perspiration), moderate (not exhausting, light perspiration), and vigorous (heart beats rapidly, sweating) PA behavior that lasted at least 10 minutes per session in the past month. The PA guidelines established by the 2008 Physical Activity Guidelines for Americans [26] which have also been recommended for cancer survivors by the American Cancer Society [27] and the American College of Sports Medicine [28] suggest that individuals obtain 75 minutes of vigorous aerobic PA per week, 150 minutes of moderate aerobic PA per week or an equivalent combination. Therefore, “PA minutes” was computed using moderate minutes plus two times the vigorous minutes. Four categories were then computed based on the guidelines for PA minutes: (1) completely inactive (no PA minutes), (2) insufficiently active (1–149 PA minutes), (3) within guidelines (150 to 299 PA minutes), and (4) above guidelines (≥ 300 PA minutes).

##### Theory of planned behavior variables

Prior to completing the TPB measures, we defined regular PA for participants as “moderate intensity PA (e.g., brisk walking) performed for at least 150 minutes per week (2.5 hours), OR vigorous intensity PA performed at least 75 minutes per week (1.25 hours).” These definitions were based on the public health PA guidelines. The TPB items were developed based on guidelines recommended by Ajzen [14,17], as well as previous studies with cancer survivors [10,11].

##### Intention

Intention was assessed by two items. The first item, “Do you intend to do regular PA over the next month,” was rated on a 7-point Likert scale from 1 (*strongly intend*) to 7 (*no, not really*). The second item, “How motivated are you to do regular PA over the next month,” was rated on a 7-point Likert scale from 1 (*not at all motivated*) to 7 (*extremely motivated*). Cronbach’s alpha (α) coefficients for internal consistency for this scale was 0.94.

##### Attitude

Attitude was measured by four items using a 7-point bipolar adjective scale that taps both instrumental (beneficial/harmful, important/unimportant) and affective (enjoyable/unenjoyable, fun/boring) aspects of attitude. The verbal descriptors were *extremely* (Points 1 and 7), *quite* (Points 2 and 6), and *slightly* (Points 3 and 5). The stem that preceded the adjectives was: “I think that for me to participate in regular PA over the next month would be…”. Separate scores for affective and instrumental attitudes were computed as they were applied as separate variables for analyses. Cronbach’s alpha (α) for the instrumental and affective attitude subscales were 0.77 and 0.81, respectively.

##### Subjective norm

Subjective norm was measured by three items rated on a 7-point Likert scale. The two items that measured injunctive norm were preceded by the stem: “I think that if I participated in regular PA over the next month, most people who are important to me would be…” followed by the scales 1 = *extremely discouraging* to 7 = *extremely encouraging*, and 1 = *extremely unsupportive* to 7 = *extremely supportive*. There was one item tapping into descriptive norm, which was “I think that over the next month, most people who are important to me will themselves participate regularly in PA” (1 = *strongly disagree* to 7 = *strongly agree*). Cronbach’s alpha (α) for injunctive norm was 0.91.

##### Perceived behavioral control

PBC was determined by two items on a 7-point Likert scale based on the guidelines from Rhodes and Courneya [29,30] that motivation should be held as a positive constant when measuring PBC. The specific items were: (a) “If you were really motivated, how much control would you have over doing regular PA over the next month” (1 = *very little control* to 7 = *complete control*); (b) “If you were really motivated, how confident would you be that you could do regular PA over the next month?” (1 = *not at all confident* to 7 = *extremely confident*). Cronbach’s alpha (α) for this scale was 0.83.

##### Underlying accessible beliefs

Underlying accessible beliefs were solicited for behavioral, control beliefs, and normative beliefs using six open-ended questions. For behavioral beliefs, participants were asked “What would be the most important benefits for you if you participated in a regular PA program and what would make PA fun or enjoyable for you (list up to three each).” For control beliefs, participants were asked to list “what factors make it easier or more difficult for you to stick with a regular PA program.” In terms of normative beliefs, participants were asked “which people or groups that are important to you would support you participating in a regular PA program or currently do regular PA themselves.”

##### Planning

Planning was measured using four items rated using a 7-point Likert scale ranging from 1 (no plans) to 7 (detailed plans) [31]. The items were: (1) “I have made plans concerning ‘when’ I am going to engage in regular PA over the next month;” (2) “I have made plans concerning ‘where’ I am going to engage in regular PA over the next month;” (3) “I have made plans concerning ‘what’ kind of regular PA I am going to engage in over the next month;” and (4) “I have made plans concerning ‘how’ I am going to get to a place to engage in regular PA over the next month.” Cronbach’s alpha (α) for this scale was 0.97.

#### Data analyses

All statistical analyses were performed using PASW Statistics 19 (PASW Inc., Chicago, IL) and AMOS 19.0 (Small Waters Corp., Chicago, IL). Descriptive statistics were calculated to determine the distribution of the variables. Bivariate correlations were computed to examine the relationship between TPB variables and PA intention and behavior. The underlying accessible TPB beliefs of the sample were determined by calculating frequencies and percentages for each of the behavioral, normative, and control beliefs. The most common underlying beliefs were reported based on the premise that each belief was solicited from at least 10% of the sample.

SEM with maximum likelihood estimation was used to allow for both an assessment of overall model fit and statistical significance tests for the size of each theoretical relation in the model (i.e., TPB). The measurement and structural models were constructed separately. For latent concept specification, the loading for each concept's first indicator was pre-set to 1.0 in the model to create a metric scale. For the single item indicators (i.e., descriptive norm, PA), a fixed error estimate of 10% and 25% was assigned to descriptive norm and PA, respectively. Model fit was assessed using a number of indices, including chi-square index, goodness-of-fit index (GFI), adjusted goodness-of-fit (AGFI), root mean square of approximation (RMSEA) and comparative fit index (CFI). While a non-significant chi-square result (*p* > .05) indicates that the model is a good fit, it is too sensitive to sample size [19], as a result additional measures are often used. GFI and AGFI scores range from 0 to 1, a score exceeding .9 indicates a good fit. RMSEA of .08, .05 and 0 indicates adequate, close and exact fits, respectively [32]. CFI and IFI have a model acceptability cut-point of > .94 [32].

When the theory underlying the model indicates that a moderating relationship among predictors may vary by specific population sub-groups (e.g., gender, age, months since diagnosis, disease stage), multi-group structural equation modeling (MSEM) using a series of models, starting from unrestricted to fully constrained is recommended [33]. A chi-square index, goodness-of-fit index (GFI) evaluates a set of complex models - one for each group. Before the invariance models are estimated, it must be established that the model is without any invariances (i.e., a model that is different in each group) is acceptable. The constraints were placed in a sequence of nested models: Model 1 was the unrestricted model: noninvariant, unconstrained model (no constrains at all) where the relationships between variables are allowed to vary as a function of the proposed moderator and will be used as a basis for comparison; Model 2 was the measurement equivalent model: equal factor loading across the sub-groups (additional constraints that the interrelationships of attitude, subjective norm, and PBC would be equal across groups); Model 3 included Model 2 constraints plus equal factor variance and covariances (additional constraints that the interrelations of attitude, subjective norm and PBC would be equal across all groups and all factor variances); Model 4 included Model 3 constraints plus equal paths (additional constraints that the interrelations of attitude–intention, subjective norm–intention and PBC–intention, PBC–behavior and intention–behavior would be equal across all groups); Model 5 included Model 4 constraints plus equal factor residuals (“fully constrained”). Models 4 and 5 examined the latent construct level, and takes into account the hypotheses about how the sub-groups may differ and are similar, in terms of their perception of variables' relationships. Therefore, the most parsimonious model that does not vary significantly from the unrestricted model was used when comparing the paths [19].

Traditionally, evidence of invariance is determined using the χ² difference test (Δχ²), however this test represents an excessively stringent test of invariance [19]. There are various ΔGFIs that are superior to Δχ² as tests of invariance because they are independent of both model complexity and sample size, and are not correlated with the overall fit measures. To compare the models, change in CFI (ΔCFI) was used [34]. Cheung and Rensvold [34] proposed critical values to indicate measurement invariance, which are robust for testing multi-group invariance. A ΔCFI ≤ -.01 indicates that the null hypothesis of invariance should not be rejected.

## Results

### Descriptives

Flow of participants through the study has been presented elsewhere [4]. In brief, of the 1,985 mailed surveys, 331 were returned to sender due to wrong address, no history of kidney cancer, or deceased. Based on the remaining 1,654 surveys, 703 were returned, generating a 35.4% completion rate (703/1,985) and a 42.5% response rate (703/1,654). For the present analyses, we had 651 of 703 (92.6%) KCS provide evaluable data for the TPB analyses.

We previously compared responders (n = 703) and nonresponders (n = 1,282) and found no differences in terms of age, sex, or surgery rate [4]. Compared to nonresponders, however, responders were approximately one year closer to their date of diagnosis, had a slightly higher rate of treatment with systemic therapy, and less likely to have renal cell carcinoma and more likely to have clear cell carcinoma [4].

Demographic and medical information for the entire sample of 703 are outlined elsewhere [4]. For the 651 participants who completed TPB data, the mean age was 64.4 ± 10.9, 62.4% were male, 79.1% were married, and the mean BMI was 28.6 ± 5.2. The mean number of months since diagnosis was 68.6 ± 56.0, 87.1% were disease-free, 97.5% had received surgery, and 83.3% had localized kidney cancer. Overall, 179 (27.4%) were meeting public health PA guidelines. Descriptive statistics and bivariate correlations for the TPB variables are reported in Table 1. 

**Table 1:** Descriptive statistics and correlations among the Theory of Planned Behavior variables in kidney cancer survivors

**Variable**	**1**	**2**	**3**	**4**	**5**	**6**	**7**	**8**	**Mean**	**SD**
1. Affective attitude	-								5.02	1.27
2. Instrumental attitude	0.60***	-							5.68	1.16
3. Descriptive norm	0.34***	0.32***	-						5.05	1.65
4. Injunctive norm	0.42***	0.54***	0.37***	-					5.90	0.96
5. Perceived behavioral control	0.40***	0.58***	0.24***	0.43***	-				4.78	1.56
6. Intention	0.55***	0.63***	0.33***	0.43***	0.69***	-			4.25	1.83
7. Planning	0.42***	0.50***	0.27***	0.34***	0.54***	0.78***	-		3.73	2.11
8. Physical activity categories	0.30***	0.34***	0.15***	0.19***	0.40***	0.50***	0.47***	-	1.89	1.65

Note: *** *p* < .001.

Physical activity (PA) categories: [1] completely sedentary (0 PA minutes), [2] insufficiently active (1–149 PA minutes), [3] within guidelines (150 to 299 PA minutes, and [4] above guidelines (≥ 300 PA minutes).

### Evaluation of the measurement and structural models

The measurement model provided a good fit to the data based on the fit statistics [χ² = 147.80, p < 0.001; TLI = 0.96; CFI = 0.98; RMSEA = 0.07, 90% CI = 0.06-0.08]. The measurement model also suggested good measurement of all the TPB constructs with significant factor loadings (p < .001). Assessment of normality was conducted to examine multivariate kurtosis. The multivariate kurtosis value represented by Mardia’s coefficient was above the recommended value of 3 [19]. Consequently, the Bollen-Stine bootstrap procedure was used to test model fit and bias corrected regression coefficients are reported for the structural model [19]. While the Bollen-Stine p-value was significant (χ² = 256.88, p < .001), other fit indices suggested that the structural model was an adequate-to-good fit to the data [TLI = 0.97; CFI = 0.98; RMSEA = 0.06, 90% CI = 0.05-0.06].

### Associations of the theory of planned behavior with intention and physical activity

**Figure 1:**
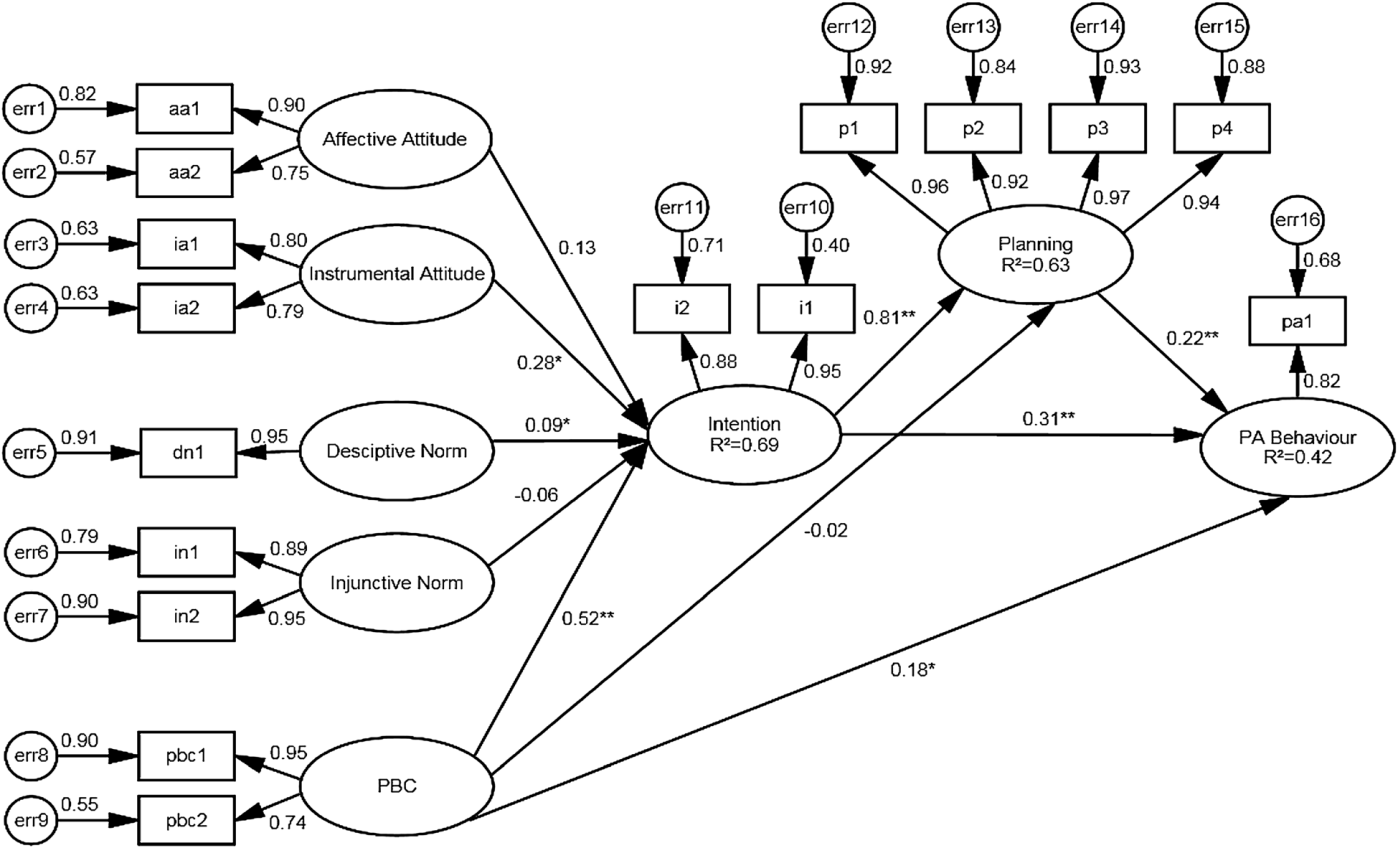
Standardized parameter estimates for pathways among the Theory of Planned Behavior in kidney cancer survivors.

Standardized, direct effect coefficients for the associations of the TPB variables on intention and PA are shown in Figure 1. There were significant pathways to PA from PBC (ß = 0.18, p = 0.02), planning (ß = 0.22, p < 0.01), and intention (ß = 0.31, p < 0.01). There were significant pathways to planning from intention (ß = 0.81, p < 0.01). In addition, there were significant model pathways to intention from instrumental attitude (ß = 0.28, p = 0.03), descriptive norm (ß = 0.09, p = 0.01), and PBC (ß = 0.52, p < 0.01). Due to non-normality, bootstrap standard errors can be larger than would be expected under normal theory assumptions, thereby influencing the significance level in the model pathways. Therefore, a larger beta coefficient may be less significant than a smaller beta coefficient [19]. 

Moreover, there were strong significant total effects of PBC (ß = 0.43, p < 0.01) and intention (ß = 0.49, p < 0.01) on PA. There were also significant total effects of instrumental attitude (ß = 0.14, p = 0.02), descriptive norm (ß = 0.04, p = 0.01), and planning (ß = 0.22, p < 0.01) on PA. In terms of the indirect effects on PA, PBC had the strongest indirect effect on PA (ß = 0.25, p < 0.01). There were also small indirect effects from descriptive norm (ß = 0.04, p < 0.01), instrumental attitude (ß = 0.14, p = 0.02), and intention (ß = 0.18, p < 0.01) on PA. Overall, the TPB accounted for 69%, 63%, and 42% of the variance in intention, planning, and PA behavior, respectively.

### Testing invariance of selected demographic and medical variables

Table 2 provides the goodness of fit indices for selected demographic and medical variables for the multi-sample nested models. The structural model was tested separately for selected demographic variables including gender (males vs. females) and age (<60 years vs. 60–69 years vs. ≥70 years). In both variables and across groups, the model provided an adequate-to-good fit to the data based on the AGFI, RMSEA, and CFI fit statistics. Given that the models offered a good fit for the sub-samples, a MSEM was conducted to determine which parameters could be considered invariant across groups. By examining the differences between the constrained models and the unconstrained models in both gender and age, the ΔCFI was ≤ -.01, indicating that the factor loadings, factor variances and covariances, interrelations between attitude, subjective norm, and PBC, planning, intention and PA behavior, and factor residuals are invariant.

**Table 2:** Goodness of fit indices for multi-sample nested models in kidney cancer survivors in Alberta, Canada

**Model**	**n**	**χ²**	***df***	***p***^***a***^	**AGFI**	**RMSEA**	**CFI**	**ΔCFI**
**Demographic variables**								
Gender								
Male	406	238.32	86	0.001	0.89	0.07	0.97	-
Female	245	184.23	86	0.010	0.87	0.07	0.97	-
Model 1		422.54	172	<0.001	-	0.05	0.97	-
Model 2		430.74	180	<0.001	-	0.05	0.97	<0.01
Model 3		453.33	195	<0.001	-	0.05	0.97	<0.01
Model 4		463.49	205	<0.001	-	0.04	0.97	<0.01
Model 5		486.38	219	0.001	-	0.04	0.97	<0.01
Age								
<60 years	246	243.51	87	<0.001	0.84	0.09	0.95	-
60-69 years	196	149.55	87	0.095	0.87	0.06	0.98	-
≥70 years	209	146.73	87	0.116	0.87	0.06	0.98	-
Model 1		539.79	261	<0.001	-	0.04	0.97	-
Model 2		567.94	277	<0.001	-	0.04	0.97	<0.01
Model 3		616.18	307	<0.001	-	0.04	0.97	<0.01
Model 4		665.14	323	<0.001	-	0.04	0.96	0.01
Model 5		801.81	353	0.001	-	0.04	0.95	0.02
**Medical variables**								
Body mass index								
Healthy	161	125.66	87	0.196	0.86	0.05	0.99	-
Overweight	281	190.35	87	0.004	0.88	0.07	0.97	-
Obese	209	161.20	87	0.038	0.86	0.06	0.97	-
Model 1		477.21	261	0.002	-	0.04	0.98	-
Model 2		500.79	277	0.003	-	0.04	0.98	<0.01
Model 3		571.39	307	0.001	-	0.04	0.97	0.01
Model 4		593.54	323	0.001	-	0.04	0.97	0.01
Model 5		693.75	353	0.014	-	0.04	0.96	0.02
Number of comorbidities								
<3	327	216.76	86	0.001	0.88	0.07	0.97	-
≥3	324	196.18	86	0.002	0.88	0.06	0.98	-
Model 1		412.94	172	<0.001	-	0.05	0.97	-
Model 2		423.41	180	<0.001	-	0.05	0.97	<0.01
Model 3		449.88	195	<0.001	-	0.05	0.97	<0.01
Model 4		464.45	205	<0.001	-	0.04	0.97	<0.01
Model 5		522.58	219	0.002	-	0.05	0.97	<0.01
Months since diagnosis								
<60	324	213.04	86	0.003	0.88	0.07	0.97	-
≥60	327	180.98	86	0.007	0.90	0.06	0.98	-
Model 1		394.02	172	<0.001	-	0.05	0.98	-
Model 2		399.41	180	<0.001	-	0.04	0.98	<0.01
Model 3		423.62	195	<0.001	-	0.04	0.98	<0.01
Model 4		435.68	205	<0.001	-	0.04	0.98	<0.01
Model 5		505.22	219	0.002	-	0.05	0.97	0.01
Type of surgery								
Partial nephrectomy	115	136.21	86	0.149	0.81	0.07	0.97	-
Radical nephrectomy	520	234.71	86	<0.001	0.92	0.06	0.98	-
Model 1		370.92	172	0.001	-	0.04	0.98	-
Model 2		386.20	180	0.002	-	0.04	0.98	<0.01
Model 3		401.98	195	0.002	-	0.04	0.98	<0.01
Model 4		411.37	205	0.002	-	0.04	0.98	<0.01
Model 5		431.09	219	0.028	-	0.04	0.98	<0.01
Type of incision								
Laparoscopic	194	197.71	86	0.005	0.83	0.08	0.96	-
Open cut	441	173.14	86	0.012	0.93	0.05	0.99	-
Model 1		370.84	172	<0.001	-	0.04	0.98	-
Model 2		386.76	180	<0.001	-	0.04	0.98	<0.01
Model 3		421.54	195	<0.001	-	0.04	0.97	0.01
Model 4		429.54	205	<0.001	-	0.04	0.97	0.01
Model 5		466.30	219	0.015	-	0.04	0.97	0.01
Disease stage								
Localized	542	242.62	86	0.004	0.91	0.06	0.98	-
Metastatic	109	130.53	86	0.098	0.80	0.07	0.98	-
Model 1		373.15	172	<0.001	-	0.04	0.98	-
Model 2		383.43	180	<0.001	-	0.04	0.98	<.01
Model 3		415.43	195	0.001	-	0.04	0.98	<0.01
Model 4		442.56	205	<0.001	-	0.04	0.97	0.01
Model 5		505.53	219	0.001	-	0.05	0.97	0.01

*Note.* Model 1-unrestricted model: noninvariant, unconstrained model; Model 2 measurement equivalent model- equal factor loadings; Model 3-model 2 constraints plus equal factor variance and covariances; Model 4-model 3 constraints plus equal paths; Model 5-model 4 constraints plus equal factor residuals (“fully constrainted”).

ΔCFI = Change in comparative fit index. A value of ΔCFI ≤ .01 indicates that the null hypothesis of invariance should not be rejected.

^a^Bollen Stine p-value reported due to multivariate non-normality.

KCS that indicated “don’t know” to type of surgery and incision were excluded from the analysis.

In addition, the structural model was tested separately for selected medical variables including BMI (healthy vs. overweight vs. obese), number of comorbidities (<3 vs. ≥3), months since diagnosis (<60 vs. ≥60), type of surgery (partial vs. radical), type of incision (laparoscopic vs. open cut), and disease stage (localized vs. metastatic). These sub-groups were created based on meaningful cut-points that are considered important targets in PA interventions, and have been used in previous studies in the cancer population [35-38]. The models for all of the medical variables represented adequate-to-good fit to the data based on the AGFI, RMSEA, and CFI fit statistics. The ΔCFI was ≤ -.01 between the constrained and unconstrained models, indicating that the factor loadings, factor variances and covariances, interrelations between attitude, subjective norm, and PBC, planning, intention and PA behavior, and factor residuals are invariant in all of the medical variables listed above.

It is important to note that age and BMI did not achieve a ΔCFI was ≤ -.01 for Model 5 suggesting that the variances and covariances of the measurement errors are not invariant across the groups. However, the testing of Model 5 is considered an excessively stringent test of multigroup invariance because measurement error variances are rarely constrained equal across groups [19].

### Most common accessible beliefs

Table 3 presents the most common behavioral, control, and normative beliefs of KCS. The nine most common behavioral beliefs regarding the advantages of PA were: (a) lose weight, (b) improve fitness, (c) improve strength, (d) feel good/better about oneself, (e) improve energy levels, (f) improve health, (g) increase flexibility, (h) improve sleep quality, and (i) lower blood pressure. The nine most common behavioral beliefs regarding what makes PA fun/enjoyable were: (a) exercise with other people, (b) exercise outdoors for fresh air/scenery, (c) do an activity that is fun/enjoyable, (d) do a variety of activities, (e) participate in team sports, (f) exercise to music, (g) exercise in good weather, (h) seeing results/benefit, and (i) do an activity that is pain-free. The 9 most common control beliefs regarding barriers to PA were: (a) other medical/health problems, (b) lack of time, (c) pain/soreness, (d) fatigue/too tired, (e) other commitments, (f) long work hours, (g) poor weather conditions, (h) lack of motivation, and (i) limited or no access to recreation facilities. The eight most common normative beliefs regarding important people that support PA involvement were: (a) family members, (b) spouse/partner, (c) friends, (d) recreation club/teammates, (e) coworkers, (f) medical team, (g) neighbors, and (h) church group.

**Table 3:** Most common behavioral, control, and normative beliefs of kidney cancer survivors in Alberta, Canada

**Beliefs**	**n**	**% Survivors**^**1**^	**% Respondents**^**2**^
			**(n = 482)**
*Most Common Behavioral Beliefs*			
* Benefits (n = 419)*			
Lose weight	207	31.8	49.4
Improve fitness	110	16.9	26.3
Improve strength	105	16.1	25.1
Feel good/better about oneself	100	15.4	23.9
Improve energy levels	95	14.6	22.7
Improve health	91	14.0	21.7
Increase flexibility	15	2.3	3.6
Improve sleep quality	14	2.2	3.3
Lower blood pressure	8	1.2	1.9
* Fun/Enjoyable (n = 334)*			
Exercise with other people	197	30.3	47.0
Exercise outdoors for fresh air/scenery	41	6.3	9.8
Do an activity that is fun/enjoyable	28	4.3	6.7
Do a variety of activities	23	3.5	5.5
Participate in team sports	22	3.4	5.3
Exercise to music	22	3.4	5.3
Exercise in good weather	14	2.2	3.3
Seeing results/benefit	15	2.3	3.6
Do an activity that is pain-free	10	1.5	2.4
* Most Common Control Beliefs (Barriers) (n = 482)*			
Other medical/health problems	115	17.7	23.9
Lack of time	104	16.0	21.6
Pain/soreness	98	15.1	20.3
Fatigue/too tired	94	14.4	19.5
Other commitments	90	13.8	18.7
Long work hours	77	11.8	16.0
Poor weather conditions	70	10.8	14.5
Lack of motivation	66	10.1	13.7
Limited or no access to recreation facilities	38	5.8	7.9
* Most Common Normative Beliefs (Support) (n = 409)*			
Family members	275	42.2	67.2
Spouse/partner	230	35.3	56.2
Friends	145	22.3	35.5
Recreation club/teammates	20	3.1	4.9
Coworkers	16	2.5	3.9
Medical team	13	2.0	3.2
Neighbors	7	1.1	1.7
Church group	7	1.1	1.7

^1^Percentage of response from all participants (N = 651).

^2^Percentage of responses from participants who answered to the questions.

## Discussion

This study is the first to examine the correlates of PA in KCS and the first to use SEM to test a two-component model of the TPB for PA in any cancer survivor group. The TPB model demonstrated an adequate-to-good fit to the data. There were significant model pathways to PA from PBC, intention, and planning, where intention emerged as the strongest correlate. In terms of planning, there was a significant pathway to planning from intention. In addition, there were significant model pathways to intention for which PBC was the strongest correlate followed by instrumental attitude and descriptive norm. Overall, the TPB accounted for 69%, 63% and 42% of the variance in intention, planning and PA, respectively. These findings are in line with previous TPB studies with cancer survivors where 21-38% of the variance was accounted for in PA behavior and 23-62% in PA intention [7-13,21,39], as well as with a recent meta-analysis in the general population where 43.7% and 21.2% of the variance was accounted for in PA intention and behavior, respectively [40]. With regards to planning, our study findings are in line with previous studies where 67% of the variance was explained by the TPB in young adult cancer survivors [8], and 71% of the variance was explained in colorectal cancer survivors [7].

In our study, PBC, intention, and planning were direct correlates of PA in KCS. The majority of studies in cancer survivors have demonstrated that intention is one of the main predictors of PA behavior [7,8,12,21], however, few of these studies have included planning. Our analyses suggest that the association of intention with PA is partially mediated by planning. A number of previous studies in the general population have also shown planning to mediate the impact of intentions on behavior and to contribute to additional variance to the prediction of behavior [17,34,41-43]. Within cancer populations, there is some evidence to suggest some implied mediation of planning for the intention-behavior relationship, where planning demonstrated independent contributions to PA among bladder cancer survivors [13], colorectal cancer survivors [7], and young adult cancer survivors [8]. This highlights that intenders may potentially benefit from formulating detailed plans to engage in PA.

Previous studies have also shown that PBC is a direct correlate of PA [13,39], however, these studies have not included planning. Our data suggest a direct association of PBC with PA even after accounting for planning. In addition, there were strong significant total effects of PBC and intention on PA. This finding may be due to age-related barriers that KCS may experience since they tend to be older than survivors of other cancers. Therefore, they may have other existing comorbidities that may contribute to poorer health. This suggests that PBC is an important correlate of PA in older populations including cancer survivors. Moreover, intention was found to be the sole direct correlate of planning which is consistent with the few studies that have examined the correlates of planning in cancer survivors [7,8]. This suggests that forming an intention is a necessary condition for the development of a detailed plan to initiate PA.

With regards to intention, the key correlates in our study were PBC followed by instrumental attitude and descriptive norm. These data suggest that KCS will form intentions to engage in PA if they view it to be easy to perform, beneficial, and that important others will perform the behavior. Moreover, when examining the indirect effects of the TPB constructs on PA, PBC had the strongest indirect effect, with descriptive norm, instrumental attitude, and intention having smaller trivial effects on PA. Similarly, previous studies in cancer survivors have also found PBC and instrumental attitude to be significant correlates of intention, with PBC being the strongest correlate [7,8,12,13,21,39]. In our study, affective attitude did not emerge as a significant correlate of intention, which is inconsistent with our hypothesis and previous research that suggests that affective attitude is a strong correlate of intention [7,8,12,13,21,39]. This finding is unique because it suggests that instrumental attitude may be more important for KCS when forming an intention to engage in PA. This may be due to differences in health and age. KCS are more likely to be overweight or obese, and have other comorbidities due to their older age compared to many other survivor groups. Therefore, KCS may be more likely to intend to engage in PA if they feel it would be beneficial rather than fun/enjoyable.

Subjective norm is typically a very weak correlate of intention after controlling for attitude and PBC [16]. In our study, descriptive norm emerged as a significant correlate of intention, but the direct effect of descriptive norm on intention was trivial, with the indirect effect on PA being small and trivial as well. Subjective norm has generally not been a significant correlate of intention in previous studies [12,21,39]. This suggests that enlisting important others to engage in PA behavior themselves and enlisting support and encouragement may not be as important among KCS compared to other TPB constructs such as attitude and PBC, or it may also indicate that normative constructs have their influence on PA through other TPB constructs (e.g., PBC, instrumental attitude, affective attitude).

A secondary purpose of this study was to examine if the TPB operated equivalently across sub-groups which consisted of common demographic and medical variables. In terms of demographic variables, the interrelationships of the TPB constructs with intention and PA behavior were invariant across age groups and sex. Similarly, invariance was also observed for medical sub-groups such as BMI, number of comorbidites, months since diagnosis, type of surgery, type of incision, and disease stage. Our finding of invariance is inconsistent with previous studies with cancer survivors that have found select demographic and medical variables to moderate associations within the TPB [13]. For example, Karvinen et al. [12] found that age and BMI moderated the associations of the TPB, where control constructs were more important correlates of PA and intention in older and obese endometrial cancer survivors compared to younger and healthy/overweight survivors. In addition, Karvinen et al. [13] found age and adjuvant therapy to be significant moderators of the TPB with bladder cancer survivors. The discrepancies in findings may be due to the differences in statistical techniques employed. In previous studies examining moderators of the TPB among cancer survivors, path analysis and multiple regression techniques were used, whereas in our study, we employed a more powerful multivariate technique of SEM which tests the TPB model overall, rather than coefficients individually [19]. These differences may also be due to the medical and demographic differences among cancer survivor groups. Our findings suggest that PA interventions for KCS based on the TPB do not need to be targeted to specific subgroups.

Our study also solicited the underlying behavioral, normative, and control beliefs for future PA interventions in KCS. The analyses of individual beliefs provide an understanding of key targets for the development of interventions designed to increase PA levels. Behavioral beliefs were separated into instrumental and affective beliefs, which is a novel feature of the elicitation of salient beliefs in cancer survivor groups. For instrumental beliefs, KCS reported weight loss, improved fitness, and improved strength as the most common anticipated benefits of PA. These findings are similar to other cancer survivor groups including young adult [8], adolescent [39], ovarian [21], endometrial [12], and non-Hodgkin lymphoma [10] cancer survivors. For affective beliefs, KCS indicated that exercising with other people, exercising outdoors, and doing a specific activity are aspects that make PA enjoyable. These beliefs are also consistent with a previous study in young adult cancer survivors [8]. Targeting these key beliefs in PA interventions is essential when attempting to influence affective and instrumental attitudes of KCS.

In terms of control beliefs, KCS reported other medical/health problems, lack of time, and pain/soreness as the most common barriers to PA. These beliefs were also reported in other cancer survivor groups [8,10,12,21,39]. Similar to our findings, Karvinen et al. [12] reported poor health to be the most common barrier to PA among endometrial cancer survivors. Given the high obesity rate and the number of comorbidites present in older cancer survivors, it is important to develop PA programs that are appropriate for people with poor health. Since PBC has been shown to be a strong correlate of intention and PA, and contribute to both total and indirect effects on PA, it is essential for PA interventions to focus extensively on control beliefs in KCS.

For normative beliefs, KCS reported that family members, spouse/partner, and friends to be the most important people to provide support. This is in line with previous research with other cancer survivor groups [8,10,12,21,39]. With older cancer survivors such as endometrial [12] and ovarian [24], family, spouse/partner, and the medical team are important sources of support which is consistent with our findings among KCS. Even though descriptive and injunctive norm had trivial and/or non-significant effects on intention, it may be important to include support and encouragement in PA interventions for KCS because of their potential influence on other TPB constructs (i.e., PBC, instrumental attitude, affective attitude).

Our study should be interpreted within the context of important strengths and limitations. To the best of our knowledge, our study is the first to examine the correlates of PA in KCS and one of the first to use SEM to examine the TPB for PA in any cancer survivor group. This study is also one of the few studies that have tested a two-component model of the TPB among cancer survivors and included planning. Furthermore, we sampled all KCS diagnosed between 1996 and 2010 from a comprehensive Registry in Alberta, Canada. One limitation of our study is the inherent selection biases due to the transparent purpose of the study. KCS who were more interested in PA were perhaps more likely to participate in the study, and thus overestimate the number of KCS meeting PA guidelines and have higher scores on the TPB variables. The modest response rate of a 42.5% may also limit the generalizability of the findings. The study design was cross-sectional in nature in which causation cannot be implied. Our study also relied on a self-report measure of PA which, although validated, can introduce measurement error.

In conclusion, our results support the utility of the TPB to explain PA among KCS. Our study provided evidence that PA is strongly associated with planning and intention which, in turn, are strongly associated with PBC, instrumental attitude, and descriptive norm. Our findings identified important targets for informing PA interventions among KCS. These interventions would need to implement strategies in regards to planning for PA and how to anticipate and overcome barriers to PA. Also, strategies can be used to address attitudes toward PA, where messages can be focused around the benefits of PA and factors that would make participating in PA important. In addition, salient PA beliefs were identified that are essential to the development of PA interventions. Based on these beliefs, PA interventions should target the benefits of PA such as weight loss and improvement in fitness and strength. The enjoyable aspects of PA should also be highlighted including exercising with others, engaging in a fun activity, and exercising outdoors. However, addressing barriers to PA such as the presence of health problems and pain/soreness, as well as lack of time should be the main target for influencing PA levels of KCS. Finally, demographic and medical variables remained invariant in the TPB model suggesting that similar intervention strategies can be implemented among different subgroups of KCS. Developing theory-driven PA interventions for KCS may lead to important improvements in health and QoL.

## Competing interests

The authors have no conflict of interest to disclose.

## Authors’ contributions

LT and KSC contributed to the conception and design of the study, acquisition of data, data analysis and interpretation, and drafted the manuscript and revised it critically for intellectual content. RCP, RER, and SN contributed to data analysis and interpretation, and have been involved in drafting the manuscript and revising it critically for intellectual content. All authors have read and approved the final manuscript.
